# Effects of bipolar disorder on maternal and fetal health during pregnancy: a systematic review

**DOI:** 10.1186/s12884-023-05924-8

**Published:** 2023-08-28

**Authors:** Malak A. Mohamed, Abdulrahman Elhelbawy, Maria Khalid, Latifa A. AbdAllatif, Hagar E. Lialy

**Affiliations:** 1 Faculty of Medicine, Helwan University, Cairo, Egypt; 2 Students’ Medical Advanced Research Team (SMART), Cairo, Egypt; 3 Faculty of Physical Therapy, Cairo University, Giza, Egypt

**Keywords:** Bipolar, Mania, Depression, Pregnancy, Maternal health, Fetal health, Obstetric Complications

## Abstract

**Background:**

Bipolar disorder (BD) is a mental disorder characterized by mood shifts from severe depression to mania. Pregnant women with BD may experience manic or depressive episodes, so they are usually concerned about the effects of BD on their pregnancy. The aim of this systematic review is to determine the effects of BD on maternal health and fetal health, weight, and development. It also addresses how BD affects the probability of incidence of pregnancy complications in women with bipolar compared with healthy controls.

**Methods:**

Seven electronic databases (Ovid MEDLINE, Embase, MIDRIS, APA PsychINFO, Scopus, Web of Science, and ScienceOpen) were searched, and 1728 eligible studies were identified. After deduplication, screening, and manual search processes, we included only 15 studies. Descriptive analysis, and calculation of the probability of incidence for each pregnancy outcome were used to analyze the results.

**Results:**

The findings of the included studies suggest that BD during pregnancy may affect both fetal growth and maternal health by increasing the risk of giving birth to an infant with some birth defects such as microcephaly, CNS problems, small for gestational age, and other congenital anomalies, in addition to causing some obstetric complications such as gestational hypertension, preterm labor, need for assisted delivery, hospital readmission, and others.

**Conclusion:**

Bipolar disorder during pregnancy negatively affects mothers and their fetuses and increases the probability of incidence of obstetrics complications.

**Supplementary Information:**

The online version contains supplementary material available at 10.1186/s12884-023-05924-8.

## Background

Bipolar Disorder (BD) is a serious mood disorder of uncertain etiology characterized by alternating manic, hypomanic, and depressive episodes. These mood swings shift from extreme highs (mania) to extreme lows (depression) and usually occur over several days or weeks. Sometimes people with BD experience mania and depression in the same episode, in which they feel energized yet depressed and hopeless. BD has two subtypes, Bipolar I and II. The 2 subtypes overlap in the basic symptoms, except that bipolar I causes full manic episodes more than bipolar II, which is characterized by hypomania that is less serious than mania and with no psychotic features [1]. Furthermore, depressive episodes are typically more frequent and long-lasting than manic or hypomanic episodes in both bipolar I and II subtypes [2–5].

BD is ranked the sixth leading cause of disability, morbidity, and mortality among people [6]. In 2005, the average annual incidence of BD’s first episode reached 5 cases per 100,000 population [7]. According to a systematic analysis of the Global Burden of Disease (GBD) in 2017, BD affects about 45 million people worldwide [8], whereas, in GBD 2019, 40 million people were suffering from BD [9]. BD affects women and men equally [10], but the cycle of illness progresses more commonly and rapidly in women [11]. In addition, women are more likely than men to have bipolar II and not adhere to their psychotic treatment [12, 13]. The onset of BD tends to occur more in adulthood between 18 and 30 years, with an average age of onset of 21 years [11].

The National Comorbidity Survey Replication reported that at least half of all BD cases start before age 25, which is mostly a period of pregnancy and childbearing in women [14]. A previous study showed that 49.8% of the women with BD I and 42.2% of women with BD II had a mood episode during pregnancy or the postpartum period [15]. Pregnancy and postpartum involve healthy processes that mothers’ bodies are prepared to carry out [16]. Being a pregnant woman with a severe mood disorder such as BD may disrupt these processes and lead to exacerbated bipolar symptoms, causing an increased risk of maternal and fetal complications.

This study aims to review the previous literature on BD and its effects on pregnancy, report the effects, risks, and obstetric complications of BD on pregnant women and their fetuses, and calculate the probability of incidence of pregnancy outcomes in women having BD.

## Methodology

### Search strategy

We conducted a database search via OVID online search interface to retrieve all relevant studies among MEDLINE, Embase, MIDRIS: Maternity and Infant Care, and APA PsychINFO. We also searched Scopus, Web of Science, and ScienceOpen databases. A unique search strategy was designed for each database according to its requirements and filters, using combinations of keywords and Boolean operators (Supplementary File 1).

### Selection criteria

Identifying our PECO and keywords (P; pregnant women) (E; bipolar disorder, affective psychosis, mania) (C; healthy pregnant women) (O; childbirth, pregnancy, delivery, labor, and parturition risks, complications, and outcomes,) we excluded studies of women with other psychological problems, BD in non-pregnant women, mixed groups of BD and other disorder, and papers addressing only the effects of lithium and other antipsychotics, whereas full-text cohort studies in English language and all settings and countries were eligible for inclusion.

### Data extraction

Data from the 15 included studies were extracted by the authors in a table of characteristics (Table 1) of 7 columns, including first author and date of publication, aim, study design, sample size, length of follow-up, country, and if there is any pharmaceutical treatment.

**Table 1:** Data extraction

First author, year	Aim of study	Study design	Sample	Pharmaceutical treatment	Length of follow-up	Country
Robert Bodén, 2012 [22]	To investigate the risks of adverse pregnancy and birth outcomes for treated and untreated bipolar disorder during pregnancy.	Population-based retrospective cohort	332,137 women: treated (n = 320), untreated bipolar (n = 554), healthy women (n = 331,263)	mood stabilizers (lithium, antipsychotics, or anticonvulsants)	1/1/2005 till 31/12/2009	Sweden
Hsin-Chien Lee, 2009 [21]	To investigate low birthweight, preterm births, and small-for-gestational-age among women with bipolar disorder or schizophrenia compared with women with no history of mental illness	Nationwide population-based retrospective cohort	A total of 528,398 singleton births between 2001 and 2003 were included; 337 were diagnosed with bipolar disorder	No specific information on medication available	between 2001 and 2003	Taiwan, China
Elad Mei-Dan, 2014 [20]	To explore the self-reported risk of adverse perinatal outcomes among women with bipolar I during pregnancy	Population-based retrospective cohort	Women with bipolar disorder (n = 1859) or major depressive disorder (n = 3724), each compared with women without mental illness (n = 432,358).	No specific information on medication available	2003 to 2011	Ontario, Canada
Katherine L Wisner, 2019 [28]	Impact of bipolar disorder on pregnancy and neonatal outcomes.	Prospective cohort	174 mother-infant dyads: BD without psychotropic treatment (BD-NP, n = 38), BD with psychotropic treatment (BD-P, n = 49), or no major mood disorder (Comp, n = 87).	Exposure to drugs within BD-P group: antidepressants (N = 62), antipsychotics (N = 55), anticonvulsants (N = 28), benzodiazepines (N = 20), lithium (N = 13), and other psychotropics (N = 9).	From 20 weeks of gestation till birth	USA
Thinh N. Nguyen, 2012 [27]	To evaluate the obstetric and neonatal outcomes of pregnant women with severe mental illness (SMI) who attended a multidisciplinary antenatal clinic in Perth, Western Australia.	Retrospective case-note audit study	138 women: Schizophrenia (n = 44), BD (n = 56), and Non-psychotic SMI* (n = 38) compared with the Western Australia population (WA) (n = 29,805)	No specific information on medication available.	Between December 2007 and April 2011	Western Australia
Assen V. Jablensky, 2005 [30]	To determine the frequency, nature, and severity of 25 obstetric complications in women with affective disorders and those with no psychiatric disorder	Retrospective cohort	763 women with BD, 1301 pregnancies; 1831 women (3129 pregnancies) with no history of mental health difficulties (controls)	No specific information on medication available.	Data of pregnancy and birth from 1980 to 1992	Western Australia
Jacqueline Frayne, 2019 [26]	To describe 10 years of antenatal care and outcomes for women with a severe mental illness (SMI)	Retrospective cohort	178 women with BD compared with 33,458 Western Australian (WA) pregnant women	Pharmacotherapy not reported.	10-year records (2007–2017)	Western Australia
Signe Heuckendorff, 2021 [29]	To determine the association between maternal mental health status and perinatal health outcomes	Registry-based prospective cohort	40,435 infants of mothers with minor mental health conditions vs. 29,919 infants of mothers with moderate-major mental health conditions (532 in BD subgroup) vs. 950,772 in reference group	At least two prescriptions of antidepressants/ benzodiazepines was identified as minor mental health condition (n = 15,120)	Between 2000 and 2016.	Denmark
Nathalie Auger, 2020 [34]	To study the association of mood disorders around pregnancy with the future risk of cardiovascular disease	Longitudinal cohort	Bipolar group = 5359 vs. Control group = 1,005,539	Pharmacotherapy not reported.	23 years of follow-up after the fifth year postpartum	Canada
M. Camille Hoffman, 2020 [23]	To determine if women with BD have higher healthcare charges and utilization of care at delivery and at 2 years post- partum, for themselves and their newborns than women with other or no mental health diagnoses	Retrospective cohort	women with BD (n = 77) compared with women with no mental illness (n = 3900) and other mental health diagnoses	No specific information on medication is available	From January 1, 2011, through December 31, 2012	USA
Aimina Ayoub, 2018 [31]	To determine if a history of mental illness in women was associated with the risk of having an infant with a central nervous system defect	population-based retrospective cohort	women with BD (n = 2432) compared with women with no mental disorder (n = 634,153)	No specific information on medication is available.	Between 1989 and 2013	Canada
Tuija Männistö 2015 [24]	To assess the effect of anxiety disorder, bipolar disease, depression, unspecified psychiatric disorder, comorbid conditions, and schizophrenia on the occurrence of preterm birth.	Observational study, retrospective cohort	206,996 No psychiatric disorders versus 515 BD pregnancies	No information about treatment.	follow-up between 2002–2008	USA
Dorothy Sit 2013 [25]	To evaluate the relationship between abnormal GDM screens, adverse outcomes, and maternal mood disorders.	Prospective cohort	61 on Health control and 45 BD	Some had been using Marijuana, (27 − 36%) on citalopram, escitalopram, fluoxetine, sertraline, and nortriptyline, (29/125 = 23%) on olanzapine, quetiapine, risperidone, prednisone, sertraline, venlafaxine, and nortriptyline, and 11% (14/125) on lithium, olanzapine, quetiapine, risperidone, and prednisone.	During time of pregnancy	USA
Aimee K 2017 [33]	To address the effect of psychotropic-exposed infants of women in the BD + group on the neuromotor performance during infancy.	Prospective cohort	Participants included 81 BD+/BD − and 116 control mother-infant pairs for a final sample of n = 197.	Two groups of BD women, one exposed to pharmacotherapy and the other don’t	Between 2007 to 2011	USA
Morgan V. 2012 [32]	To examine risk of intellectual disability and other neuropsychiatric outcomes in children of mothers with BD compared with unaffected mothers.	Retrospective Cohort	Bipolar group n = 763, compared with control group n = 1831	No specific information on medication is available.	Data of children born between 1980–1992	Western Australia

### Risk of bias assessment

All the included studies are cohort studies (5 prospective and 10 retrospective studies); the risk of bias in the 15 studies was assessed using New Castle Ottawa (NOS) assessment tool for cohort studies [17] (Table 2).

**Table 2:** Risk of bias (NOS) of the 14 included studies

First author, year	Selection Domain				Comparability Domain	Outcome Domain			Overall Quality
	Representativeness of the exposed cohort (Max.★)	Selection of the non-exposed cohort (Max.★)	Ascertainment of exposure (Max.★)	Demonstration that outcome of interest was not present at start of study (Max.★)	Comparability of cohorts on the basis of the design or analysis controlled for confounders (Max.★★)	Assessment of outcome (Max.★)	Was follow-up long enough for outcomes to occur (Max.★)	Adequacy of follow-up of cohorts (Max.★)	
Robert Bodén, 2012 [22]	**-**	★	★	-	★	★	★	★	**Fair**
Hsin-Chien Lee, 2009 [21]	-	★	★	-	★★	★	★	-	**Fair**
Elad Mei-Dan, 2014 [20]	-	★	★	-	★★	★	★	**-**	**Fair**
Katherine L Wisner, 2019 [28]	-	★	★	★	★★	★	★	★	**Good**
Thinh N. Nguyen, 2012 [27]	-	★	★	-	★	★	★	-	**Fair**
Assen V. Jablensky, 2005 [30]	-	★	★	-	★	★	★	★	**Fair**
Jacqueline Frayne, 2019 [26]	-	★	★	-	★	★	★	★	**Fair**
Signe Heuckendorff, 2021 [29]	-	★	★	★	★	★	★	**-**	**Good**
Nathalie Auger, 2020 [34]	-	★	★	★	★★	★	★	★	**Good**
M. Camille Hoffman, 2020 [23]	-	★	★	-	★★	★	★	★	**Fair**
Aimina Ayoub, 2018 [31]	-	★	★	-	★★	★	★	★	**Fair**
Tuija Männistö 2015 [24]	-	★	★	-	★★	★	★	**-**	**Fair**
Dorothy Sit 2013 [25]	-	★	★	★	★★	★	★	**-**	**Good**
Aimee K 2017 [33]	-	★	★	★	★★	★	★	**-**	**Good**
Morgan V. 2012 [32]	-	★	★	-	★★	★	★	-	**Fair**

### Data analysis

The total number of women in the included studies’ bipolar and control groups was 4,243,334 pregnant women. Most of the studies were conducted in USA (n = 5), then Western Australia (n = 4), Canada (n = 3), Sweden (n = 1), China (n = 1), and Denmark (n = 1).

## Results

### Search results

**Fig. 1:**
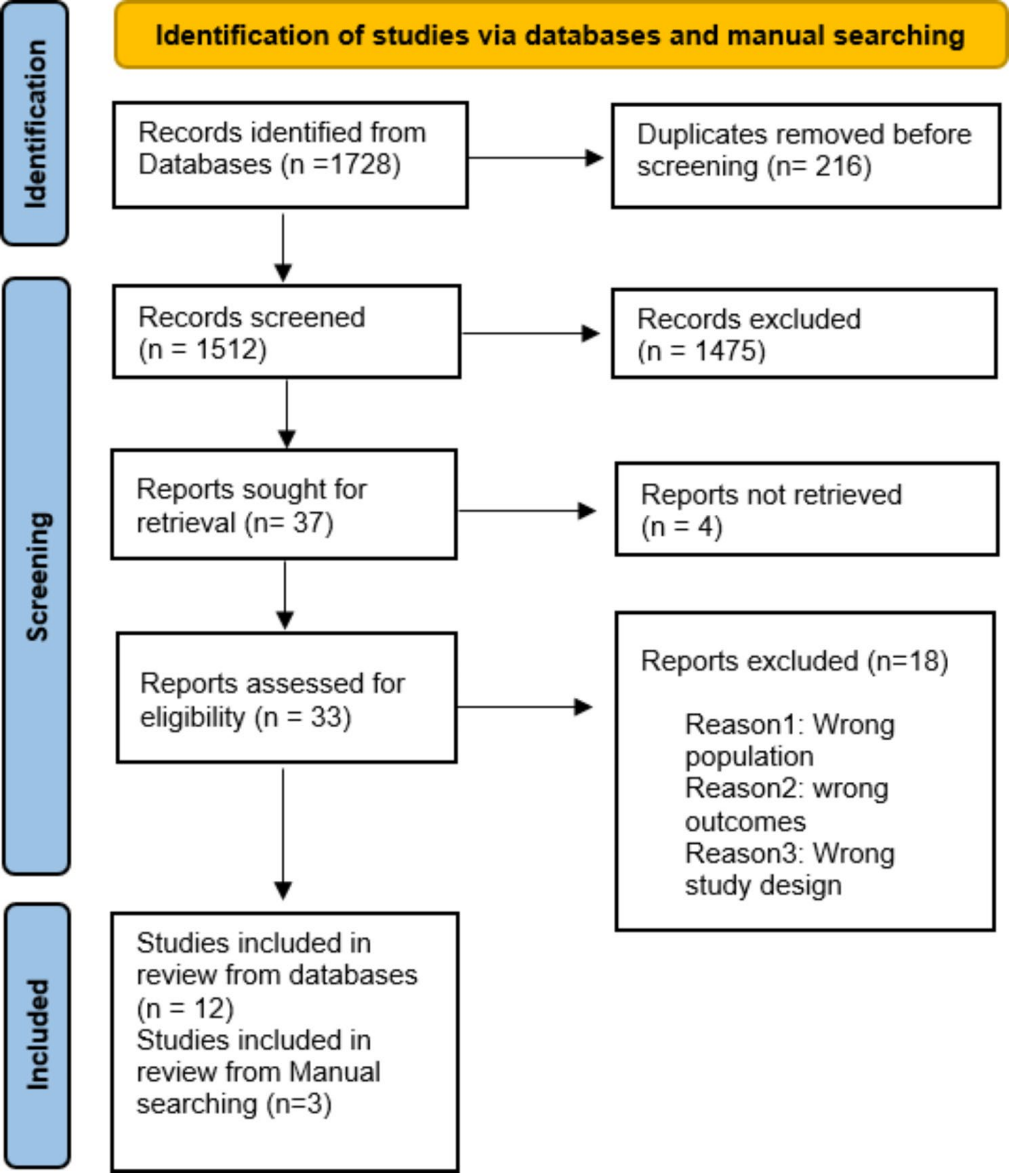
PRISMA Flowchart

The search yielded a total of 1728 studies: OVID 4 databases (n = 1332): MEDLINE, Embase, MIDRIS, and APA PsychINFO, Scopus (n = 663), Web of Science (n = 101), and ScienceOpen (n = 10). We imported each database results file into EndNote software [18] for deduplication. After removing duplicates (n = 216), 1512 studies were obtained for title-and-abstract screening. Each 2 authors screened the studies independently, and conflicts were resolved by discussion. According to our inclusion/exclusion criteria, we excluded 1475 papers, and 37 studies were sought for retrieval. Only 33 of them have full-text free access. The full-text screening was done, and only 12 papers were eligible for inclusion from the 7 databases, and 3 papers were obtained from manual search. Figure 1 shows our PRISMA flowchart [19].

### Qualitative analysis

Due to the heterogeneity of the data, all the studies including 30 outcomes were analyzed using qualitative synthesis.

#### Preterm birth (PTB) < 37 weeks

7 studies [20–26] showed that bipolar disorder increases the risk of preterm birth, however, 2 studies [27, 28] showed that there is no significant difference between bipolar and control groups.

Mei-Dan et al. [20] found that the prevalence of PTB was higher in women with BD (11.4%) than in the comparison group (6.2%). Furthermore, Lee et al. [21] found that women with BD were more likely to have PTBs than pregnant women with no history of mental illness (14.2% vs. 6.9%); the odds of PTB for women with BD were 2.08 higher than healthy women after maternal, paternal, and infant characteristics were considered. Boden et al. [22] also found that the risk of PTB significantly increased for women with BD whether treated (8.1%) or untreated (7.6%), compared with women without BD (4.8%). Moreover, Hoffman et al. [23] found that women with BD were significantly (p < 0.001 “clinically significant”) more likely to have more than twice the rate of preterm birth infants compared with women with no mental health diagnosis (15.5% vs. 6.9%, respectively). Furthermore, Männistö et al. [24] reported that all psychiatric disorders have a significant effect on preterm birth between 37 and 34 weeks’ gestation, especially BD; preterm birth occurred in 10.3% of BD group versus 7.5% in the healthy controls. Sit et al. [25] also reported 14% PTB incidence in BD group, compared with 6.5% in healthy controls, which was the lowest between all groups. Another study [26, 29] also shows a higher prevalence of PTB with women having BD (12.4%), compared with WA mothers (7.5%), (p < 0.001 “clinically significant”).

However, Nguyen et al. [27] reported no significant difference in PTB in infants of women with BD, compared with infants of WA mothers (8.9% vs. 7%, P = 0.548). Wisner et al. [28] also reported that the proportions of late preterm births were similar across the 3 groups: treated BD (8.2%), untreated (5.3%), and comparison group (6.9%) and that the risk remained non-significantly different, adjusting the potential confounding effects of maternal age, race, employment status, use of illicit drugs, and pre-pregnancy BMI; approximately 10% of all participants’ infants were delivered preterm overall. reported that all Psychiatric disorders have a significant effect on Preterm birth between 37 and 34 weeks’ gestation, it was 15,485 (7.5%) among 206,996 on no psychiatric disorders group versus 53 (10.3%) among 515 pregnancies (0.2%) with BD group.

#### PTB < 32 weeks and < 28 weeks

Both Mei-Dan et al. and Männistö et al. [20, 24] showed that bipolar increases the risk of PTB < 32 weeks. Mei-Dan et al. [20] found that PTB < 32 weeks increased in the BD group, compared with the referent group (9.1% vs.1.1%), while no significant increased risk in PTB < 28 weeks was reported between BD (0.9%) and the referent group (0.6%). Männistö et al. [24] also reported that PTB < 34 weeks, especially < 28 weeks, occurred more often to women with BD, compared with those with no psychotic disorder (8.3% vs. 3.7%, respectively).

#### Small for gestational age (SGA) < 10th percentile

Three papers reported an association between having BD and giving birth to SGA infants. Hoffman et al. [23] found that women with BD were more likely to have an earlier gestational age at delivery, compared with women with no mental health diagnosis. Mei-Dan et al. [20] found that women with BD have an increased risk of SGA < 10th percentile, compared with women without mental illness: BD: 14.1%, reference group 12.8%. AOR = 1.17 (95%CI 1.03–1.34), adjusted for maternal age and parity only. Lee et al. [21] also reported that women with BD were more likely to have SGA than healthy pregnant women (22.3% vs.15.7%).

#### Severe SGA < 3rd percentile

The three studies, Mei-Dan et al., Boden et al., and Jablensky et al. [20, 22, 30], showed that there is no difference between both bipolar and control groups in severe SGA.

Mei-Dan et al. [20] found that the risk of severe SGA was not significantly elevated in the bipolar group (4.6%) vs. women with no mental difficulties (3.9%), AOR (1.15; 95% CI, 0.92–1.43) when adjusted for maternal age, income quintile, parity, infant sex, obesity, substance abuse hypertension, venous thromboembolic disease, and gestational diabetes mellitus (DM), gestational hypertension (HTN), preeclampsia/eclampsia. Boden et al. [22] also showed that there are no significant differences for severe SGA reported between women with BD (neither treated with mood stabilizers nor not) and the control group, as the birth weight for untreated = 3.4%, treated = 2.5%, and no BD = 2.3%, birth length for untreated = 3.8%, treated = 3.2%, and no BD = 2.3%, and head circumference (HC) for untreated = 3.9%, treated = 3.3%, and no BD = 2.3%. This study shows that untreated BD was associated with the most increased risk of SGA regarding weight, length, and HC. After adjusting for confounders, the increased risks were somewhat attenuated and no longer statistically significant except for microcephaly, as the infants of women with untreated BD were at an increased risk of microcephaly than others. Furthermore, Jablensky et al. [30] defined the SGA as less than 37 weeks. The authors found no significant difference in SGA occurrence between women with BD and those with no mental illness (6.2% Vs 7.6%), as the mean gestational age (weeks) was 39.1 in the BD group, whereas 39.0 in the comparison group.

#### Large for gestational age (LGA)

Mei-Dan et al. [20] divided the LGA into LGA > 90th percentile and severe LGA > 97 percentile: In LGA > 90th percentile, the authors noticed no significant difference in occurrence between women with BD (9.1%), compared with women with no BD (8.2%), AOR 1.13 95% CI 0.96 − 0.32, adjusted for maternal age and parity only. Regarding severe LGA > 97th percentile, it was more common among women with BD than in the control group (3.8% vs. 2.7%, AOR = 1.29; 95% CI, 1.08–1.54). Boden et al. [22] also measured severe LGA and found no significant results between women with treated and untreated BD (2.5% vs. 2.3%). Overall, women with bipolar disorder (treated or not) were at an increased risk of having an LGA infant for weight than the control group, although this was not statistically significant. Treated women were at a non-significantly increased risk of having an LGA infant for length. Untreated women were less likely to have LGA for length. It seems that the infants of treated women with BD will not develop either macrocephaly or microcephaly, whereas women with untreated BD are more likely to develop microcephaly, but not macrocephaly. Furthermore, Frayne et al. [26] defined LGA at > 37 weeks and > 4000 g, and they found that there was no significantly increased risk of LGA for infants of women with BD, compared with the WA population (7.1% vs. 11%, respectively, p-value = 0.285).

#### Congenital malformations

Jablensky et al. [30] found no difference in congenital abnormalities, defects of the cardiovascular system, and cerebral palsy, a rare complication of the neonate, in infants of women with BD compared with those of women with no mental health difficulties. However, Mei-Dan et al. [20] found that BD caused a significant risk of congenital malformations in infants of the BD group (RR between BD and control is 1.4). Furthermore, Boden et al. [22] suggest that psychotic treatment during pregnancy causes a high risk of congenital malformations, as the prevalence of congenital abnormalities was higher for infants of women with BD who are treated with mood stabilizers than those born to women with untreated BD or without BD (3.4% vs. 1.9% vs. 2%).

#### Central nervous system (CNS) defects and head circumference (HC)

Three studies [22, 28, 30] found that the HC was smaller in the newborns of mothers with BD than in those of mothers with no mental health difficulties; two of them [22, 28] reported that the incidence rate of small HC was higher in unmedicated women with BD, compared with women undergoing bipolar treatment. Ayoub et al. [31] also found that women with BD had 2.49 times the risk of having an infant with a CNS defect, compared with those with no mental illness. Among specific nervous system defects, they found that women with BD was most strongly associated with microcephaly (RR 3.70, 95% CI 3.21–4.26), compared with healthy women. They also had a higher risk of hydrocephalus (1.28 (1.03–1.60)), after adjusted regression models.

Furthermore, Morgan et al. [32] reported an increase in the intellectual disability in the offspring of maternal bipolar group, compared with those of unaffected mothers (3.1%) vs. (1.0%), respectively. The study also found that odds of epilepsy increased approximately twofold in children of the maternal bipolar group vs. control group, 1.2% vs. 0.7%, respectively. Children of the participants also experienced convulsions with 4.0% in bipolar group vs. 3.0% in comparison group, however, it remains statistically insignificant.

#### Fetal distress

Jablensky [30] et al. found that fetal distress occurred non-significantly more often in women with BD than in unaffected women (13.6% vs. 11.8%). There was no significant difference in OR before and after adjustment for maternal characteristics (1.18 vs. 1.17, respectively). However, Nguyen [27] et al. found that suspected fetal distress was significantly more common in women with BD compared to WA mothers (21.4% vs. 12.6%; P < 0.0001).

#### Low birth weight of infant < 2500 g

Lee et al. [21] found women with BD were more likely to have low birth weight infants than pregnant women with no history of mental health difficulties (9.8% vs. 5.7%). Furthermore, Hoffman et al. [23] found that women with BD were significantly (p < 0.001 “clinically significant”) more likely to have low birth weight infants, compared with women with no mental health diagnosis (20.8% vs. 6.4%, respectively). Sit et al. [25] also found that BD group’s infants have an average weight of [3,229 g -+569 g] versus [3,604 g -+555 g, p < 0.001] in the control group.

However, two studies [27, 29] found no difference in the low birthweight incidence rate or the mean birthweight between infants of women with BD and WA mothers and their babies.

Wisner et al. [28] found that the mean birth weight did not significantly differ (p = 0.079) between BD-NP and the other two groups but tended to be lower in the BD-NP group. Another study [30] showed no significant difference between bipolar and control groups having LBW infants (9.9% vs. 9.3%).

#### 5-minute apgar score < 7 or < 8

Three studies [22, 28, 30] reported no difference found in low 5-min Apgar scores between infants of women with BD and those with no mental illness. However, Nguyen et al. [27] found that babies born to women with BD attending the CAMI clinic were less likely to have an Apgar score of 8 at 1 min (64.3% vs. 85%; P < 0.0001) and an Apgar score of 8 at 5 min (89.3% vs. 97.5%; P < 0.0001) than healthy women. On the other hand, a higher percentage of Apgar score < 7/5 min. was observed in infants of BD group (1.5%) vs. those of the reference group (0.8%) by Heuckendorff et al. [29]

#### Rare syndromes of offspring and pervasive developmental disorders

Morgan et al. [32] examined the distribution of rare syndromes like Hurler, Klinefelter, Moebius, Noonan, Prader–Willi, Rett, Rubinstein–Taybi, VATER association, and Turner syndromes among offsprings of participants and found that the risk was elevated in those of mothers with BD (0.4%), compared with those of unaffected mothers (0.1%).

The study also shows that the risk of children having pervasive developmental disorders including autism was increased significantly in the maternal BD group, compared with the uneffected group, 0.3% vs. 0.0%, respectively.

#### Stillbirth

Mei-Dan et al. and Jablensky et al. [20, 30] noticed that there is no significant difference found in the rate of stillbirths between women with BD and those with no mental health difficulties.

#### Neonatal readmission at < 28 days of neonate life

Mei-Dan et al. [20] reported that BD group had an increased risk of neonatal readmission (2.0%), compared with the control group (0.9%). Heuckendorff [29] et al. also found a higher risk of neonatal readmission at BD group (15.2%) than control group (11.3%). However, Wisner et al. [28] found no significant differences in the neonatal intensive care unit (NICU) admission occurred across the three groups: BD with treatment = 4.1%, untreated BD = 7.9%, and comparison = 8.1%. The risk remained non-significantly different across the three groups (p = 0.729) in the multiple regression models adjusting for the potential confounding effects of maternal characteristics, but it was noticed that women taking treatment for bipolar are less likely to get their babies readmitted to NICU, compared with both the non-treated and healthy women.

#### Neonatal hypoglycemia

Only one study [22] reported infants of women with untreated BD were at a higher risk of neonatal hypoglycemia (4.3%) than treated (3.4%) BD women and women without BD (2.5%), but the risk estimates in treated BD were imprecise.

#### Neonatal morbidity

Mei Dan et al. [20] defined neonatal morbidity as respiratory distress syndrome (RDS), seizures, sepsis, intravenous hyperalimentation (IVH), persistent fetal circulation, and neonatal abstinence syndrome. The risk of having any of these neonatal morbidities was higher in women with BD (5.4%) compared with those without mental illness (1.9%) AOR 2.99, 95% CI 2.44– 3.66). When analyzed separately, babies of women with BD had higher rates than the referent group for RDS: 1.5% vs.1.0%, seizures: 0.6% vs. 0.2%, sepsis: 1.3% vs. 0.7%, IVH: 0.7% vs. 0.3%, and neonatal abstinence syndrome: 1.9% vs. 0.0% (great difference), respectively. However, no difference was seen between the two groups in the incidence rate of persistent fetal circulation.

#### Infant mortality

Two studies [20, 30] reported that there was no difference in neonatal mortality (< 28 days) or postnatal death (< 1 year) in the BD group compared with women with no mental illness group; one of them [26] also showed no difference in childhood death between the 2 groups.

#### Motor quality and psychomotor development

Aimee et al. [33] showed that infants of BD+ mothers were more likely to have less motor quality score on components of the Bayley Scale of Infant Development II (BSID-II) - Behavioral Rating Scale. BD+ group’s infants were mostly less than the 75th percentile at the 52-week developmental assessment, as only 11.5% of them were assessed as above the 75th percentile, compared with 40.0% in the BD− group and 48.4% in the nonexposed group.

#### Labor or delivery obstetric complications

Jablensky et al. [30] found that there was no difference in the incidence rate of cephalopelvic disproportion, atypical presentation, cord anomalies, postpartum hemorrhage, early rupture of the membranes, prolonged labor, and threatened abortion between women with BD and unaffected women. Women with pre-existing BD had more risk of obstetric complications than those who developed BD after the index birth.

#### Gestational diabetes Mellitus (GDM)

Boden et al. [22] noticed no increased risk for GDM between either pharmaceutically treated BD, untreated BD, and control groups, with no difference in odds ratio even after adjustment for smoking, and diagnosis of alcohol or substance misuse disorder. Mei-Dan and Lee et al. et al. [20, 21] also found no significant difference in rates of GDM between women with and without BD. However, Frayne [26] et al. reported that women with BD are more likely to have GDM than WA mothers, (10.7% vs. 7.4% respectively; p < 0.001 “clinically significant”). Nguyen [27] et al. also supported this finding; they found that women with BD attending the CAMI clinic were at an increased risk of developing GDM than WA mothers (12.5% vs. 4.4% respectively; P < 0.0001).

#### Gestational hypertension (GHTN) (preeclampsia)

Lee et al. [21] found women with BD were more likely to have GHTN (1.5% vs. 0.5%) than pregnant women with no history of mental health difficulties (p < 0.02). The odds ratio (AOR) was 2.81 when adjusted for maternal, and infant characteristics (95% CI 2.53–3.10). However, Mei-Dan [20] found no difference in GHTN incidence rate between BD and control groups.

Another three studies showed a great impact of BD on GHTN high prevalence; Frayne et al. [26] found a higher prevalence of GHTN in women with BD (15.7%), compared with WA mothers (2.1%), (p < 0.001 “clinically significant”), Nguyen et al. [27] found that women with BD had an increased risk of GHTN than WA mothers (10.7% vs. 2.7%, respectively; P < 0.0001) and Jablensky et al. [30] reported that pregnant women with BD were more likely to have preeclampsia than pregnant women with no history of mental illness, (12.5% vs. 10.9%, unadjusted OR 1.17, 95% Cl = 0.93—1.48), but the authors did not mention the adjusted OR, which may differ from the unadjusted one).

#### Antepartum hemorrhage (APH)

Jablensky et al. [30] showed that Australian women with BD were more likely to have APH than other women (4.2% vs. 2.6%). It was noticed that it is a specific and significant complication in women with pre-existing BD not who developed it after the index birth. Compared with WA mothers (2.8%), Frayne et al. [26] also found a higher prevalence of APH in women with BD (9%, p < 0.001 “clinically significant”). However, Nguyen et al. [27] found no clinically significant (P = 0.232) difference between APH incidence rates in women with BD and WA mothers.

#### Threatened preterm labor

Nguyen et al. [27] found that BD caused an increased risk of developing threatened preterm labor compared with no mental illness in the WA obstetric population (7.1% vs. 2.5% respectively; P = 0.006). Another study [26] also found that the risk of threatened preterm labor was elevated in women with bipolar, compared with WA mothers, (10.1% vs. 2.2%, respectively, p < 0.001). However, one study [30] found no significant difference in incidence between women with BD and unaffected women (4.2% vs. 3.8%).

#### Placenta previa

Only one study [30], showed that women with BD were more likely to have placenta previa (1.6%) than pregnant women with no BD (0.8%, unadjusted OR 2.04 95% CI 1.11–3.73). Placenta previa was also noticed to be specific in the BD group.

#### Time to spontaneous respiration and intubation

No difference was found in time to spontaneous respiration > 2 min and intubation in women with BD compared with those with no mental health difficulties [30].

#### Naloxone administration

Jablensky et al. [30] found that infants born to mothers with BD are more likely (3.4%) to be administrated with a narcotic antagonist (naloxone), compared with those born to healthy mothers (2.4%). The unadjusted OR was (1.44. 95% Cl = 0.99—2.11), and after adjustment for maternal age, parity, plurality, marital status, aboriginality, and sex, the odds remained significant (AOR 1.38; 95% CI 0.94—2.02).

#### Cesarean section (CS)

Boden et al. [22] found that women without BD had a rate of cesarean birth of 16.8%, while both untreated and treated women with bipolar had increased risks of 23.5% vs. 25.6%, respectively. Männistö et al. [24] also reported an increase in prelabor CS in BD (14.4%) vs. control group (11.5%).

However, Frayne et al. [26] found that there was no difference in emergency and elective CS rates between the women with BD and WA mothers.

Nguyen et al. [27] found that women with BD are less likely to choose elective CS than WA mothers (12.5% vs. 17.9% respectively, P = 0.954), but a non-significant difference in emergency CS was found.

#### Instrumental delivery (assisted delivery)

Two studies found that BD caused an increased risk of assisted delivery [22, 27]; one of them [22] reported that women without BD had a rate of instrumental birth of 24.7%, while both untreated and treated women with bipolar disorder had increased risks (33.0% vs. 34.1%, respectively). The other study [27] also found that infants of women with BD are more likely to be born through instrumental delivery than WA mothers (28.6% vs. 13.7%, respectively, P = 0.019). Furthermore, Frayne et al. [26] found that assisted delivery rates between the women with BD and WA mothers were 20.2% vs. 15.1%, respectively, P = 0.085).

#### Non-spontaneous start of labor (induction of labor)

Boden et al. [22] found that BD caused high rates of the non-spontaneous start of labor, as the rate in women with no mental illness was 20.7%, while both untreated and treated women with BD had increased risks of 30.9% vs. 37.5%, respectively, whereas sit at al [25]. showed {174/515= (33.8%)} induced labor among BD group vs. {72,064 /206,996= (34.8%)} in no psychiatric disorders group.

#### Perinatal events

Sit et al. [25] identified perinatal events as preterm births and complications such as neonatal care admissions, pre-eclampsia, feeding difficulty, transient respiratory distress, and low Apgar scores at 1 and 5 min, and others. The authors measured these stressful perinatal events with peripartum events scale (PES) and found that when it came to the incidence of any (PES ≥ 1) of the previously mentioned perinatal events, the control group (24.6%) showed a higher incidence than BD (19.1%), but for the increased perinatal events (PES ≥ 2) incidence, BD showed a higher incidence (6.4%) than control group (4.9%).

#### Cardiovascular hospitalization

Auger et al. [34] discussed the association between BD in women with their risk of developing cardiovascular diseases before, during, and after pregnancy; the authors found that generally, BD was associated with a higher cumulative incidence of cardiovascular events (3.0 times) than no mental illness group. This incidence increased over time. Compared with the reference group, women with BD had 3.4 times the risk of heart failure, 2.4 times the risk of myocardial infarction, and 3.1 times the risk of pulmonary embolism. However, BD was not associated with ischemic stroke and heart procedures.

### Probability of incidence of the outcomes

Calculating the probability of incidence of each complication for bipolar and control cohorts using Bayes’ rule (P(A) = P(A∩B)/ P(B|A)) (A denotes women or fetuses having certain outcome or complication, and B denotes presence of those women or fetuses in certain sample), we found that the most significant differences in the probability of incidence between women with BD and women with no mental illness were in preterm birth (13.6% vs. 6.8%, respectively; in a total population = 1,569,849), gestational HTN (10.5% vs. 0.73%, respectively; total population = 596,325), and microcephaly (10.8% vs. 2.3%, respectively; total population = 336,567). Women with bipolar also showed a higher probability for the incidence of gestational diabetes (10.6% vs. 5.6%, respectively; total population = 69,049), threatened preterm labor (5% vs. 2.4%, respectively; total population = 67,927), and APH (4.6% vs. 3.08%, respectively; total population = 67,927) than unaffected women.

Neonates of women with BD show a higher probability of incidence of some complications, when compared with mentally healthy pregnant women’s neonates: congenital malformations (5% vs. 3.5%, respectively; total population = 446,038), low birth weight (9% vs. 0.13%, respectively; total population = 556,702), neonatal readmission (2.5% vs. 0.9%, respectively; total population = 434,991), and SGA < 10th percentile (16.4% vs. 14%, respectively; total population = 963,215).

## Discussion

This systematic review evaluates the effects of BD on pregnant women and their infants. The available data suggests that bipolar contributes to the increased risks of some maternal and fetal complications during pregnancy and delivery; preterm birth, gestational HTN and small HC (microcephaly) were the most significant outcomes in BD participants. The risk of threatened preterm labor, instrumental delivery, neonatal admission, low birth weight, SGA < 10th percentile, congenital malformations, and CNS defects also increased because of BD. The findings also suggest that congenital malformations are more likely to be found in infants of women taking psychotic treatment for BD.

However, insufficient evidence or different results were found regarding the adverse effects of bipolar on other pregnancy outcomes such as newborn length, fetal distress, SGA < 3rd percentile, LGA, Apgar score < 8, stillbirth, neonatal morbidity, neonatal mortality, neonatal hypoglycemia, induction of labor, prolonged labor, threatened abortion, placenta previa, cord anomalies, cephalopelvic disproportion, atypical presentation, spontaneous respiration > 2 min, intubation, antepartum HGE, postpartum HGE, early rupture of membrane, narcotic antagonist administration, cardiovascular maternal hospitalization, infant motor quality and psychomotor development, child’s rare syndromes, developmental disorders, epilepsy or convulsions, and others.

It was noticed that the PTB < 37 weeks was the most mentioned and repeated outcome in pregnant women with BD among most of the included studies; we interpret that as the placenta is firstly exposed to any potential physiological mechanisms that are associated with bipolar [20]. The increased risk of PTB can also be exaggerated by some confoundings and multifactorial risks such as smoking, cannabis use, and obesity. Other known factors are medical comorbidities and pregnancy complications, especially GDM and hypertensive disorders, which affect fetal growth. Another possible theory, which may interpret why PTB has the most increased risk, is that despite medical advances and efforts to improve obstetrical care, the frequency of preterm delivery remains substantially increased [25] PTB is an important adverse outcome, as it is a strong predictor of perinatal death [25].

Similar results were found in systematic reviews by Rusner et al. [35] and Depra et al. [36], which evaluated a wide range of outcomes of BD on pregnancy, that support our findings, as they found an increase in risks of preterm birth, congenital malformations, SGA < 10, and gestational hypertension.

The included studies mentioned that there are some maternal and fetal outcomes not affected by any confounding and remained significant after adjustment of maternal characteristics. This suggests that these pregnancy outcomes may be due to the direct impact of bipolar pathophysiology itself on the women and her fetus. This leads us to try to understand the potential physiological mechanisms that contribute to more adverse pregnancy outcomes in women with BD compared with women with no mental illness.

As depression symptoms increase cortisol and catecholamine secretion, hypoperfusion of the placenta occurs, thus, fetal growth and development are affected [20]. This interprets why the conditional probability of incidence of congenital malformation, SGA < 10th percentile, and LBW are higher when women have bipolar compared with when they are unaffected. The high premature growth of neonates born to women with BD explains why the probability of neonatal readmission is higher with those neonates compared with those born to women with no mental health difficulties. Indirect or direct effects of health risk behaviors and activated stress pathways [25] could be another explanation for why mood disorders in pregnancy could result in adverse outcomes. Furthermore, there may be a shared genetic susceptibility for both adverse perinatal outcomes and serious mental illness [20].

However, the studies showed variety in controlling variables, so confoundings have a role in increasing risks of some complications in women with bipolar, as this disorder may expose her to certain health disparities and lifestyle behaviors such as smoking, alcoholism, and substance misuse disorder. Sit et al. [25] found mothers with BD smoked cigarettes (16/45 = 36%) and used substances including marijuana (14/45 = 33%) significantly more often than healthy control (3/61 = 4.9% and 4/61 = 6.6%); this difference reflects that the nature of the disease is risky. It may alter stress responses in patients with BD and provoke an inappropriate release of counter-regulatory hormones, such as cortisol, and accelerated production of inflammatory markers, leading to abnormal fetal arousal, and placental dysfunction [25]; this finally ends with exaggerating risks for preterm delivery, neonatal distress and other complications.

Women with severe mental illnesses such as BD also have unstable, unhealthy lifestyles, obesity (pre-pregnancy BMI > 30) and poor diets, which may impact and elevate the risk of pregnancy outcomes. for example [25], women with high pre-pregnancy BMI are linked to gestational obesity or GDM and strongly associated with perinatal mood disorder and major depression. Both diabetes in pregnant women and perinatal mood disorders are interrelated; one leads to another. Studies show that the risk for GDM is as twice as high in depressed mothers, and lack of medical intervention for GDM may increase the risk of depression and other mood disorders such as BD. Thus, GDM or BD may have direct or indirect contribution in increasing the risk of adverse pregnancy outcomes. It is found that hyperglycemic mothers with BD during pregnancy are more likely to develop preeclampsia, delayed onset of diabetes later in life, increased risk for congenital malformations, birth weight > 90th percentile, neonatal hypoglycemia, and respiratory distress syndrome [25]. Mothers with mood disorders such as BD with GDM are also more likely to experience PTB [25]. This supports the proposed correlation between gestational obesity, perinatal mood disorders such as bipolar, GDM, and adverse pregnancy outcomes, especially PTB.

To reduce the risk of BD consequences, regular antenatal care and follow-up of pregnant mothers with BD and child for comprehensive care are encouraged to improve maternal and child health and reduce the risk of adverse pregnancy outcomes. Improving practice including increasing pregnancy screening, the need of timing intervention, and using multidisciplinary teams to provide holistic care are also recommended. This is besides involving the patient, her family members, and healthcare team for psychosocial management through a shared decision-making process. Clinicians should be aware of the increased risk of adverse pregnancy outcomes in women with affective psychosis, as some of which may be preventable [37]. Further efforts that focus on improving identification, assessment, and linkage to care for women with and at risk for BD in the perinatal period are critical to addressing the problem of perinatal complications. This includes evaluating and determining the best methodologies for screening for the illness, as well as the most effective ways to integrate BD care detection and management into obstetric settings [38].

Although this review does not focus on the effects of antipsychotic drugs exclusively and the difference between drugs on various adverse pregnancy outcomes, comparing treated and untreated groups with BD to the control group may give a hint about which outcomes are increasingly related to BD medications and which are correlated with BD pathophysiology.

Pregnant women with BD often have a prescription for lithium, antipsychotics, carbamazepine, lamotrigine, or valproate during pregnancy. Some studies found that these drugs are linked with congenital malformations, preterm birth, LGA, and GDM [36], while another systematic review of 29 studies [39] found that lithium was not related to PTB and LBW when compared with women with BD unexposed to lithium, supporting our results and hypothesis that BD pathophysiology is the major cause or preterm birth and, thus, LBW in infants of women with bipolar. Nonetheless, other studies even found a positive impact of the medications on reducing the risks of some complications.

The difference regarding these complications between those who were treated and untreated compared with the healthy group is clarified by Boden et al. [22]. Regarding low Apgar score, infants of the treated women had a less risk of a low Apgar score, while untreated women with bipolar were associated with giving birth to an infant with a low Apgar score. However, results showed no difference between increased risk for GDM in both groups, compared with those without BD. The untreated maternal bipolar was associated with an increased risk of infants being small for gestational age < 3rd percentile for weight and length, while infants of women with untreated bipolar were at a significantly increased risk for SGA for microcephaly. Another two studies [28, 30] confirmed that not treating women with BD comprises a high-risk obstetrical population with a specific risk for giving birth to newborns with smaller HC. However, these studies show that among women on psychotropic medications, the risk of having a SGA infant for weight, length, or head circumference was not statistically significantly increased. For LGA, the women who were treated probably did not develop either macrocephaly or microcephaly, but they had increased risk of LGA for weight and length, while untreated BD were more likely to develop microcephaly rather than macrocephaly, and infants had an increased risk of SGA for weight and length. These findings suggest that it is possible that the drugs used to treat bipolar, which included both antipsychotics and valproate, might have masked growth restriction by enhancing fetal growth. Further support for this hypothesis is reflected in finding increased risks of having LGA infants for weight and length in treated women with BD [22].

Mood stabilizers are also linked with increased risk of congenital malformations [36]. The incidence of treated women with BD having an infant with congenital malformation was higher than in both untreated and control groups. Another two studies [20, 30] confirmed that the women who are not being treated with mood stabilizers in pregnancy may not be at the same level of increased risk of congenital malformations. Another systematic review supports our findings [39], as it reports that lithium is responsible for the increasing risk of congenital malformations and cardiac anomalies in offspring of women with BD.

There is inconsistent evidence to support discontinuation of maintenance medications for women with BD, except for valproic acid, which is not recommended for use by pregnant women [36]. So, it is important for physicians to know the effects of these drugs and how they can manage the benefits-risks ratio for these drugs to favor women with BD.

Sometimes, lithium is chosen through the shared decision process to treat BD in pregnancy, keeping in mind its known side effects for controlling them [37]. A case report [37] mentioned that the timely intervention during the acute episodes of BD and timely management of complications can prevent avoidable obstetric and neonatal complications.

Women with a history of mental illness had a higher risk of CNS defects during subsequent pregnancies compared with women with no mental illness [31]; the strength of this association depends on the type of mental illness. Bipolar was more strongly associated with non-neural tube defects such as microcephaly. The risk of CNS defects was even greater for women diagnosed with multiple mental disorders- such as BD with other mental disorders, - hospitalized more frequently for mental disease, or first admitted at age 17 years or later. Thus, more severe mental illness before pregnancy may indicate a potentially greater risk of CNS anomalies in future offspring. The etiology of mental disorders with offspring CNS defects is complex or multifactorial, as folic acid deficiency and methylenetetrahydrofolate reductase (MTHFR) gene polymorphisms, with shared evidence of defective folate metabolism, aberrant DNA methylation, and oxidative stress may be reasons for this association. Environmental risk factors and genetic mutations may also interact and affect brain development, increasing the risk of microcephaly and hydrocephalus in infants [31]. Enhanced mental health screening in young women, timely diagnosis of BD, considering its associated risk of nervous system defects in offspring, and folic acid supplementation once mental illness is diagnosed may be beneficial to prevent CNS anomalies in offspring of women with BD.

### Strengths and limitations

The strength of this review lies in providing the probability of incidence of each pregnancy outcome between bipolar and control groups. Furthermore, assessing the quality of the studies included shows that the total scores for 5 studies were good, whereas the other 10 were assessed as fair. However, this systematic review has a limitation of language restriction.

## Conclusion

Bipolar affects maternal health, as mothers suffering from BD during pregnancy have an increased risk of some complications such as preterm birth, threatened preterm labor, gestational hypertension, and instrumental delivery. Bipolar and its psychotic treatment also have adverse effects on fetal health and development, increasing the risks of congenital malformations, small HC and CNS problems, SGA < 10th percentile, and, so, neonatal hospital readmission. However, the available data suggests that BD does not increase the incidence of stillbirth and infant mortality.

More research is needed to determine the effects of BD on cardiovascular events risk in mothers, obstetric complications such as neonatal hypoglycemia, neonatal morbidity, spontaneous respiration > 2 min, intubation, naloxone administration, neuromotor performance of infant, and occurrence of rare syndromes, developmental disorders, autism, epilepsy, and convulsions to the offspring, due to the limited number of available studies and insufficient information. Because of the inconsistent findings among the included studies regarding severe SGA, LGA, cesarean section, APH, GDM, Apgar score < 8, low birth weight, and fetal distress, conducting more studies is crucial to determine the effects of BD on these complications.

### Electronic supplementary material

Below is the link to the electronic supplementary material.

Supplementary Material 1

## Data Availability

The datasets used and/or analyzed during the current study are available from the corresponding author on reasonable request.

## List of abbreviations

BD: Bipolar Disorder
PTB: Preterm Birth
SGA: Small for Gestational Age
LGA: Large for Gestational Age
HC: Head Circumference
GDM: Gestational Diabetes Mellitus
GHTN: Gestational Hypertension
APH: Antepartum Hemorrhage
CS: Cesarean Section
CNS: Central Nervous System
BSID-II: Bayley Scale of Infant Development II
PES: Peripartum Events Scale
