# Supplementary material for: Effects of bipolar disorder on maternal and fetal health during pregnancy: a systematic review
Source: BMC Pregnancy Childbirth. 2023 Aug 28;23:617. doi: 10.1186/s12884-023-05924-8 (PMC10464164; doi:10.1186/s12884-023-05924-8)
Supplement: Supplementary file 1 — Supplementary Material 1 [file 12884_2023_5924_MOESM1_ESM.docx]

Embase <1974 to 2023 January 27>

Ovid MEDLINE(R) ALL <1946 to January 27, 2023>

MIDIRS: Maternity and Infant Care

APA PsycInfo <1806 to January Week 4 2023>

1 exp bipolar disorder/ 153389

2 exp Pregnancy/ 1792211

3 1 and 2 2731

4 remove duplicates from 3 2196

5 delivery.af. 2006840

6 pregnancy.af. 2318667

7 5 or 6 3967477

8 2 or 7 3993278

9 bipolar personality disorder.mp. [mp=ti, ab, hw, tn, ot, dm, mf, dv, kf, fx, dq, bt, nm, ox, px, rx, an, ui, sy, tc, id, tm] 23

10 bipolar*.ti. 95489

11 cyclothymia.ti. 242

12 1 or 10 or 11 176585

13 8 and 12 6240

14 exp cohort studies/ 3399324

15 cohort$.tw. 2339532

16 14 and 15 1242109

17 13 and 16 293

18 exp cohort studies/ 3399324

19 cohort$.tw. 2339532

20 controlled clinical trial.pt. 95170

21 epidemiologic methods/ 246268

22 limit 4 to yr=1966-1989 245

23 exp case-control studies/ 1602354

24 (case$ and control$).tw. 1535389

25 exp cohort analysis/ 3400974

26 exp longitudinal study/ 346854

27 exp prospective study/ 1483051

28 exp follow up/ 1963847

29 cohort$.tw. 2339532

30 exp case control study/ 1602354

31 (case$ and control$).tw. 1535389

32 18 or 19 or 20 or 22 or 23 or 24 6076553

33 25 or 26 or 27 or 28 or 29 or 30 or 31 8077045

34 32 or 33 8152978

35 13 and 34 1520

36 18 or 19 or 20 or 22 or 25 or 26 or 27 or 28 or 29 6755732

37 13 and 36 1332

38 remove duplicates from 37 1158

Scopus:

TITLE-ABS ( "bipolar disorder" ) AND TITLE-ABS ( pregnancy OR delivery OR labor OR labour ) AND ( LIMIT-TO ( SRCTYPE , "j" ) ) AND ( LIMIT-TO ( OA , "all" ) ) AND ( LIMIT-TO ( PUBSTAGE , "final" ) ) AND ( LIMIT-TO ( DOCTYPE , "ar" ) ) AND ( LIMIT-TO ( LANGUAGE , "English" ) )
